# Implementation of Positive Advanced Recovery Connections in Primary and Secondary Mental Health Care—A Registered Advanced Nurse Practitioner‐Led Initiative

**DOI:** 10.1111/jan.16498

**Published:** 2024-10-01

**Authors:** A. Cunningham, D. De La Harpe Golden, M. Pink, E. Truszkowska, T. Byrne, C. Murphy, A. Strahann, C. Doyle, A. Kasemiire, T. Frawley

**Affiliations:** ^1^ Community Healthcare East, Bray Mental Health, Bray Primary Care Centre Bray Co. Wicklow Ireland; ^2^ School of Nursing, Midwifery and Health Systems University College Dublin Belfield Dublin Ireland; ^3^ Mental Health Ireland, Marina House, Dun Laoghaire Co. Dublin Ireland; ^4^ Centre for Support and Training in Analysis and Research University College Dublin Dublin Ireland

**Keywords:** advanced practice, care pathways, clinical guidelines, delivery of health care, nurse practitioners, primary/secondary care interface, psychiatric nursing, psychosocial nursing, research implementation

## Abstract

**Aim(s):**

This study reports on the implementation of a registered advanced nurse practitioner intervention. Aims include improving access, service user outcomes and integration between primary and secondary care.

**Design:**

This paper reports the quantitative results of a mixed methods implementation study. Qualitative data are reported separately. The PARiHS framework informs the implementation process itself, with considerations for nurses and other healthcare professionals explored.

**Methods:**

The CORE‐OM 34 item rating scale was administered both pre‐ and post‐intervention. Service user attendances in secondary care was monitored.

**Results:**

Findings suggest that the intervention was associated with clinically significant improvements in global or generic distress, reported by service users, as evidenced by changes in the CORE‐OM scores. Access to care was recorded at an average of 3.6 days. Implementation science supported effective and safe implementation with clear governance structures.

**Conclusion:**

Registered advanced nurse practice in mental health clinics which provide full episodes of care results in improved integration and may be associated with positive patient outcomes. Implementation science is taught on Irish nursing programmes and this is important if innovative services are to be embedded in the healthcare system.

**Impact:**

The development of a model of care for mental health Registered Advanced Nurse Practitioners at the interface of primary and secondary care settings may be merited. Positive Advanced Recovery Connections may be associated with improving mental health outcomes and bolstering integration of primary and secondary care services. The utilisation of implementation science highlights the need for collaboration with all stakeholders to overcome barriers and recognise facilitators to attain the necessary model of integrated care.

**Patient and Public Contribution:**

Peer recovery input was provided by members of the service Recovery College, with participation evident in all stages of the project. The psychosocial assessment template was also co‐designed.

## 1 Introduction

This paper reports on the results of an evaluation of a registered advanced nurse practitioner (RANP) in mental health‐led implementation initiative. This initiative is called Positive Advanced Recovery Connections (PARC). The aim of the initiative was to improve access to evidence‐based mental health treatments for people presenting with mild to moderate mental health illnesses at the primary care level. Simultaneously, the initiative seeks to increase access to secondary care mental health services for people with severe and enduring mental health illnesses. A further aim of the initiative was to improve integration of primary and secondary care services including improved communication between all disciplines, a goal reported upon in the United States context by Harris (2023).

Access to care is a significant factor in service user feedback across settings (Gyberg et al. 2023). As well as reporting on the implementation of a RANP‐led initiative, this paper sets out a detailed description of the specific role. Additionally, much needed evidence is presented exploring how such advanced practice may impact care at the levels of individual clinical outcome and, more broadly, the health system. The need for accessible mental health services is increasingly apparent across the globe (Salami, Salma, and Hegadoren 2019), with demand on mental health services growing at primary care level (O'Connor et al. 2020). There are several reasons for this including but not limited to population growth and urbanisation (Ventriglio et al. 2021), impacts of the COVID‐19 pandemic (Keyes et al. 2023), increasing mental health literacy (Sutton et al. 2016) and efforts to address stigma (Goh, Yong, and Tam 2021). Despite these trends at primary care and policy levels, globally, initiatives to implement more comprehensive, integrated mental health services have had mixed success (Moise, Navin, and Wainberg 2021; Reeve et al. 2016). A clear gap then to be addressed is an apparent lack of streamlined communication and referral pathways between primary and secondary care services, which is also highlighted by McIntyre et al. (2022). In certain jurisdictions, a further reason may be separate funding pathways for both services.

Engaging with implementation processes, optimises the potential of advanced nursing practice, enhances understanding of the role and increases the ability to collaborate effectively across disciplines (Clarke et al. 2023). We contend there is a need for nurses and other healthcare professionals to have a knowledge of implementation science (Casey, O' Leary, and Coghlan 2018). This is particularly the case in mental health services where nurses among others have led substantial change such as deinstitutionalisation, over the last four decades (Department of Health, Ireland [DoH] 2020). This paper offers an account of how clinical innovation in mental health nursing was supported by implementation science. The PARiHS framework guided this process which is explored in the background and discussion sections. Implementation science is recommended to close the gap between research and translation to the practice environment. Findings that such innovation is associated with improvements in service user outcomes and access to care are also presented.

### 1.1 Understanding PARC

The implementation initiative is named PARC and is provided within an East Coast of Ireland Community Mental Health Team (CMHT). At the outset, PARC established a referral pathway for consultant psychiatrists and general practitioners (GPs) to access specialist mental health RANP clinics, with advanced practice nursing possessing a track record of enhancing access to care (Ramage et al. 2021). Service users attending primary care services may be referred by their GP to engage in a psychosocial assessment by the RANP, which requires an element of triage, a potentially complex decision‐making process (Jung and Yi 2023). Following assessment, service users can access specialist psychosocial interventions (cognitive behavioural therapy; the decider skills programme [group and one to one]) provided by the mental health nursing (MHN) team, alongside community signposting. The psychosocial assessment and interventions provided are outlined in more detail in Table 1. Service users attending the secondary care psychiatry service can also self‐refer for these psychosocial interventions once they have had a psychiatric assessment by the consultant psychiatrist or registrar. The RANP clinics offer an interface between the primary and secondary care setting by providing a direct link between GPs and consultant psychiatrists.

**TABLE 1:** Description of intervention.

RANP Psychosocial Assessment	The RANP practices at a higher level of capability and with that can provide full episodes of care including assessment, diagnosing, prescribing, onward referral and discharge. Psychosocial assessment is provided by the RANP who reviews the psychological, social, personal, relational and vocational needs of the person and is an evidence‐based intervention (Trenoweth and Moone 2017). The psychosocial assessment includes a comprehensive and clearly recorded mental state examination, physical health assessment, medication review, risk assessment and a review of strengths and problems in all areas of life. The assessment template was co‐produced with service users. All mental health nurses working on the programme are registered with the Nursing and Midwifery Board of Ireland.
Cognitive‐behavioural therapy (CBT)	CBT is an evidenced‐based talking therapy that focuses on how a person's thoughts, behaviours and emotions are connected and helps a person become aware of how these may be impacting their emotions. The cognitive‐behavioural model purports that our thoughts and beliefs influence our behaviour and emotions (Beck and Alford 2008). CBT is the most researched form of psychotherapy, with numerous studies demonstrating its effectiveness for a range of psychological problems.
The Decider Skills	The Decider Skills Programme provides coping skills in the event of an emotional emergency, increasing independence and resilience, reducing impulsivity and resulting in more positive outcomes for the person. The Decider Skills Programme is strongly grounded in theory and is recognised as being helpful within both the primary and secondary care setting, while also being cost‐effective (Ayers and Vivyan 2016).

PARC has allowed for the recommended principle of horizontal integration, partnering a co‐operative relationship between the primary and secondary care settings, therefore providing healthcare to service users at the appropriate level (Health Service Executive 2024). Service users can access mental healthcare from the RANP while remaining under the care of their GP. Oversight of the RANP's caseload is provided by consultant psychiatry supervision. Where indicated, the RANP in collaboration with the service user, can transfer care to the consultant psychiatrist and CMHT. To promote intervention consistency and fidelity, formal peer supervision was provided by an established advanced nurse practitioner, professional supervision was made available by the area director of nursing and a patient and public involvement group was formed with implementation science guiding the process. In the case of PARC, weekly consultation meetings for all mental health nurses were provided by the RANP, alongside monthly external CBT supervision for all practitioners delivered by a recognised British Association for Behavioural and Cognitive Psychotherapies supervisor.

A number of factors underpinned the clinical and business case for PARC. Local service evaluations indicated that waiting times for psychosocial interventions ranged from 1 to 49 weeks. Despite regular psychiatric review, when offered psychosocial interventions, 47% of service users did not attend. The RANP initially proposed a business case for PARC to the area director of nursing and the consultant psychiatrist which would allow for earlier access to psychosocial interventions. Following this, the RANP presented the proposed service to the CMHT, and the executive management team. These communications assisted with the acceptance of PARC. Regular attendance at GP clinics by the RANP and other members of the CMHT supported buy‐in and an increase in referrals. Working closely with community partners resulted in positive relationships with the knowledge that support was available should that be required. Alignment with an established clinical practice guideline (CPG), and national policy was considered an important facilitator of PARC.

For the purpose of facilitating international comparison, the RANP is a registered nurse with at least 5 years post‐registration practice. Such a practitioner will have completed master's level certification in an advanced area of clinical practice, leading to additional credentialing with the national regulator, the Nursing and Midwifery Board of Ireland (NMBI). RANPs are well placed to lead on health service improvement initiatives (Ryder, Jacobs, and Hendricks 2019) as they possess authentic clinical leadership, the importance of which is explored by Dirik and Intepeler (2023). The role and function of the RANP is to identify gaps in services, such as access to care for service user's and provide solutions to these gaps. Critical challenges faced by the health service include reducing emergency unscheduled care to the hospital or unnecessary admissions, supporting early discharge to the community or easing access to both secondary specialist and/or primary care community supports, therefore reducing waiting lists in all areas (International Council of Nurses 2020; DoH 2019; Casey et al. 2019). This is possible as RANPs can provide full episodes of care which include assessing, diagnosing, prescribing, making onward referral and discharging service users (NMBI 2017). PARC also provides interventions which can assist the service user to transition from secondary care to primary care as their illness eases from severe to moderate or mild. We hypothesise that creating a RANP‐led clinic that provides psychosocial assessment, onward referral to psychosocial interventions provided by mental health nursing and community signposting can improve service user outcomes and integrate the primary and secondary care services. The fact that the RANP can refer into secondary CMHT care with ease where required, manages identified risks in a timely and safe manner and creates a model of integrated care.

This RANP initiative is based on an existing CPG specifically *Common Mental Health Problems: Identification and Pathways to Care* (NICE 2011). Bridging the gap between secondary CMHTs and primary care services is fundamental to increasing access to specialised care and community supports (Mental Health Reform 2022; DoH 2020, 2019; Health Service Executive [HSE] 2020; National Office for Suicide Prevention [NOSP] 2020). By way of example, the role of primary care in preventing suicide in the longer term management of self‐harm is pivotal to successful outcomes. Many people who die by suicide have not been in contact with the mental health services or an emergency department (Hardy 2019). A multi‐system approach is necessary across all social and healthcare sectors with the recognition that primary care has the potential to reduce the occurrence of suicide (Firaz et al. 2021). Similarly, reducing the progression of common mental health illnesses to severe and enduring presentations is a key goal (NICE 2011). Moreover, PARC is inspired by extant national policy, ‘Sharing the Vision (2020‐2030)’ which recommends that services shall operate on an ‘integrated basis’ (HSE 2020, 73), with mental health services no longer ‘seen as a separate service within a larger structure’ (HSE 2020, 73) and cohesion being delivered as opposed to aspired for. Aligning mental health services with emerging health policies is viewed as paramount with services provided ‘across primary care, social care, mental health, and health and wellbeing in a more coordinated and integrated way’ (HSE 2020, 73). The alignment of primary and secondary care, cross boundary working, closer to the community is envisioned. Furthermore, in an Irish policy context, PARC appears to adhere to the Sláintecare principles of providing the right care, in the right place at the right time by way of RANP psychosocial assessment and improved access to evidence based interventions. Finally, the Expert Review Body on Nursing and Midwifery highlights the important role of RANPs and general practice nurses in promoting integrated care (DoH 2022).

## 2 Background

The interface between primary and secondary mental health services may be described as challenging. Such is the gap between primary, secondary and tertiary care, taking a global perspective, the World Health Organization (WHO) specifically called for building on existing human resources and mental health infrastructure to increase inputs in primary care from mental health specialists (Wang et al. 2007). Peer and Koren (2022) in an integrative review outline how poor relationships can stymie integration between primary care, secondary and tertiary mental health services. Other factors too may have an adverse impact. These include siloed documentation and national data collection responsibilities (Heslop et al. 2016), fragmentation of care and provision of care by non‐mental health staff grades (MacLeod et al. 2021) and variable experience of family members of those attending for care (McCann, Bamberg, and McCann 2015).

At the primary care level, there is scope for improvement in facilitating access to secondary care mental health specialists. Yet, despite this, there is limited focus on advanced nursing practice in primary care. There is opportunity to better understand and document the experience of implementing new advanced practice roles (Masso and Thompson 2017) in mental health nursing especially as they relate to the integration of primary, secondary and tertiary services (Wells et al. 2019). This is particularly relevant as a majority of mild to moderate mental health illness presentations arise in primary care (Gulati, Cullen, and Kelly 2021). In Ireland, government policy is to increase the number of advanced nurse and midwife practitioner roles to 2% (approximately 750 practitioners) of the overall nursing workforce to create an initial critical mass (DoH 2019). Furthermore, the Expert Review Body on Nursing and Midwifery (DoH 2022) calls for integration of care between the community and acute hospital network. Taken together, there is a clear opportunity for RANP roles to support mental health pathways between the acute and community settings.

Key ingredients in implementation science influenced PARC. It is important to determine whether policy and guidelines have been implemented effectively and therefore making data available is an essential component of this (Brownson, Chriqui, and Stamatakis 2009), which is central to the thinking of the project team. Frawley, Meehan, and De Brún (2018) pinpoint how organisational structure is an essential facet to ensuring that implementation of policy occurs. In PARC, the importance of a collaborative interdisciplinary and nursing leadership structure was apparent. A shared vision at all levels of the organisation is important from staff to executive nurse levels (Ooijen et al. 2022). Similarly, the support, engagement and clinical governance provided by other disciplines, such as psychiatry, is key. In older literature, Lugon and Seckler‐Walker (1999) outline the role of a quality and safety committee with supporting roles, functions and reporting structure. In PARC, a stakeholder group, representative of GPs, service users, family, secondary care CMHT experts and nursing leaders was essential to explore barriers or facilitators. McSherry and Pearce (2011) identify a host of factors which can impact implementation namely a lack of understanding, fear, an open or closed culture, a belief that the intervention represents nothing new, a lack of time or resources, poor leadership, a belief that it is a tool of management, a lack of support or ineffective communication. Due to the collaboration between all PARC stakeholders, these concerns did not feature; however, it is crucial that nurses and other healthcare professionals are aware of the risks inherent when implementing new care processes.

RANPs are required to engage with implementation science. It is necessary for RANPs to utilise a scientific framework to navigate an often complex healthcare system. The PARiHS framework informed the implementation of PARC and is described as a ‘multi‐dimensional framework which was developed to explicitly challenge the pipeline conceptualization of implementation’ (Bergström et al. 2020, 1; Rycroft‐Malone 2004). Evidence, context and facilitation are key elements of the PARiHS framework (Kitson, Harvey, and McCormack 1998). In PARC, these are expressed through the adaptation of evidence (a CPG developed by the National Institute for Health and Care Excellence, *Common Mental Health Problems: Identification and Pathways to Care*), context (specific features of the community mental health service which piloted PARC) and facilitation (characteristics of the RANP, nursing leadership and staff, consultant psychiatrists and other team members). Put simply, the PARiHS framework identifies the potential barriers or enablers that influence implementation process and outcomes (Rycroft‐Malone, Seers, and Wallin 2018). The framework hypothesises that for successful implementation evidence gathered from research, clinical experience, patient experience and local information must be considered (Bergström et al. 2020). The quality of the context for implementation must also be contemplated, namely the culture, leadership, the way evaluation is performed and how the implementation is facilitated within services. When these facets are considered, the nurse or healthcare professional must have regard for both the external or scientific approach and the internal or intuitive approach (Rycroft‐Malone, Seers, and Wallin 2018).

There has been continuous criticism that despite the publication of many national reports and strategy documents, several do not make it to fruition or are partially implemented even though the evidence generated remains valid, which is often described as the quality chasm (House of Oireachtas 2017; Damschroder and Lowery 2013). Any change in healthcare may be viewed as a complex bundle which involves many moving pieces. Therefore, the intervention becomes a ‘complex intervention’ which is adapted to fit the labyrinthine and dynamic healthcare setting (May 2013, 2). Provision of healthcare is provided by a socially organised system that has dynamic and contingent relations, where the agents or the individuals/groups interact with each other. The implementation process itself is a deliberate planned proposal to bring in new or modified practices which in turn change the social system (May, Johnson, and Finch 2009). When facilitators of implementation are considered, adherence to national policy, advances in treatment and a commitment to evidence‐based practice are prominent examples. Whereas resistors to successful implementation may be exclusively top‐down instruction, or a lack of consensus building with key stakeholders and opinion leaders not taking place (Braithwaite et al. 2018). The implementation of CPGs, which underpin projects such as PARC, is recognised as difficult due to the complexity of the healthcare system (Francke et al. 2008). There are core concepts of the CPG that cannot change but there are parts that may be adapted to fit the local context (Damschroder et al. 2009). The loss of fidelity must be considered with any suggested changes, that is, that the original intention of the CPG regarding positive service user outcomes is not lost (Haynes et al. 2015). Robust implementation theories can guide the implementation journey, identifying the barriers and promoting facilitators of the proposed intervention (Finch, Mair, and O Donnell 2012).

Additionally, a critical factor for any implementation is the less understood concept of context, which is described as the environment within which the intervention is being considered, a dynamic feature embracing both the physical setting and social environment (Rogers et al. 2021). A scoping review conducted by Nilsen and Bernhardsson identified common contextual factors across the literature including organisational support, financial resources, leadership, social relationships and organisational culture and climate (Nilsen and Bernhardsson 2019). A further review by Rogers and colleagues did however suggest that context, as a concept, requires ongoing investigation as it is not consistently or clearly defined within the literature (Rogers et al. 2021). One thing is clear, context and intervention implementation occur at multiple levels within the complex healthcare system and must be considered before the implementation process begins (Nilsen 2015). For the system to function all stakeholders, including service users, must be involved and included in change as equal partners. Engaging with stakeholders is fundamental to ascertain barriers or facilitators of a proposed plan (Peters, Tran, and Taghreed 2013).

## 3 The Study

### 3.1 Research Focus and Aims

Our work sets out to answer the following question: can service user outcomes be improved, and primary and secondary care services integrated, by implementing a mental health RANP‐led clinic which provides psychosocial assessment, onward community signposting and early access to available secondary care interventions? We will examine this by:

1. Assessing service user problems and symptoms, well‐being, risk and functioning both pre‐ and post‐intervention using the CORE—OM rating scale (34‐item scale).
2. Monitor access to care.
3. Determine if the number of service users presenting with mild to moderate mental health illness attending the secondary care setting, increases or decreases.

## 4 Methods/Methodology

### 4.1 Design

Our evaluation employed a pre‐ and post‐design comprising of quantitative methods of data collection. This manuscript reports on the quantitative findings only while qualitative results are reported separately. Data collection took place from June 2021 to June 2022. This study adheres to the StaRI guideline, included in Appendix S1.

### 4.2 Study Setting and Sampling

PARC is based in an urban primary care centre and is staffed by members of a CMHT. Specifically, during this data collection period, the psychosocial assessment was provided by the RANP only. Psychosocial interventions were delivered by three community mental health nurses, one senior staff nurse and two staff nurses (mental health) alongside the RANP. Additional governance is provided by two consultant psychiatrists and the nursing management team, now consisting of assistant, director and area director of nursing. All service users referred to PARC were invited to participate (*n* = 215). Of these, 157 completed both pre‐ and post‐measures, indicating a 73% response rate. Reasons for non‐engagement included college or work commitments and physical illness including COVID‐19. All those referred were offered a standardised RANP psychosocial assessment and psychosocial interventions provided by mental health nurses. Data collected were paper based and added to the clinical record. It was subsequently de‐identified and stored electronically on a password protected computer by the RANP, situated in the primary care centre.

### 4.3 Inclusion and/or Exclusion Criteria

Inclusion and exclusion criteria are listed in Table 2. Following referral, all service users were contacted by the RANP, and given the opportunity to opt in for psychosocial assessment.

**TABLE 2:** Inclusion and exclusion criteria.

Inclusion criteria	Exclusion criteria
General practitioner referral	Age under 18 years
Consultant psychiatrist referral	Age over 65 years and have not been assessed by the Mental Health Service previously
Age 18 years and upwards	Residing outside service catchment area
Mild to moderate mental health signs, symptoms or illness	Referral suggestive of a severe and enduring mental health illness diagnosis
Resident within the service catchment area	Current inpatient status

### 4.4 Instrument Validity and Reliability

The Clinical Outcomes in Routine Evaluation (CORE‐OM) was utilised both pre‐ and post‐intervention. This tool has 34 items assessing global or generic distress measures, covering areas of: well‐being (4 items) [*W*]; problems and symptoms (12 items) [*P*]; functioning (12 items) [*F*] and risks (6 items) [*R*] and includes positively and negatively framed items (Connell et al. 2007). There is also an overall CORE‐OM outcome score. A higher score on the CORE measures, domains or individual items indicates a higher level of distress or symptom severity. Evans et al. (2020, 51) report on issues of validity and consistency and find the tool is ‘a reliable and valid instrument, with good sensitivity to change’. Specifically, internal and test–retest reliability were considered good, with a range of 0.75–0.95 being reported. Additionally, there was good sensitivity to change while convergent validity with seven other instruments was outlined. Meanwhile, Barkham et al. (2006) provide an overview of how the CORE‐OM may be used in practice.

### 4.5 Data Collection and Data Analysis

The CORE‐OM was provided in person in the first and final session to participants by administrative staff. The course of assessment and interventions averaged 8 weeks. Those who disengaged were sent a stamped addressed envelope with CORE‐OM for return. The response rate was 73%, with data collected over the 12‐month period from June 2021 to June 2022. Data were analysed using R (version 4.3.1), a statistical computing software, by a statistician employed by the partner university.

### 4.6 Ethical Considerations

Ethical approval was granted on 20 April 2021 by the Service Ethics Committee with reference number 2021/1/REF. A participant information leaflet was given to service users alongside an option to consent in writing by non‐clinical administration staff. Written informed consent was obtained from all participants. Participants were aware they could opt out of the research with no adverse implications for care.

## 5 Results

The following sections namely primary care dataset, secondary care dataset and decider skills group data represent the outcomes of assessing service user problems and symptoms, well‐being, risk and functioning both pre‐ and post‐intervention using the CORE‐OM rating scale (34‐item rating scale). Meanwhile, the section additional performance indicators report on access to care and number of service users presenting with mild to moderate mental health illness within the secondary care setting.

### 5.1 Primary Care Data Set

**FIGURE 1:**
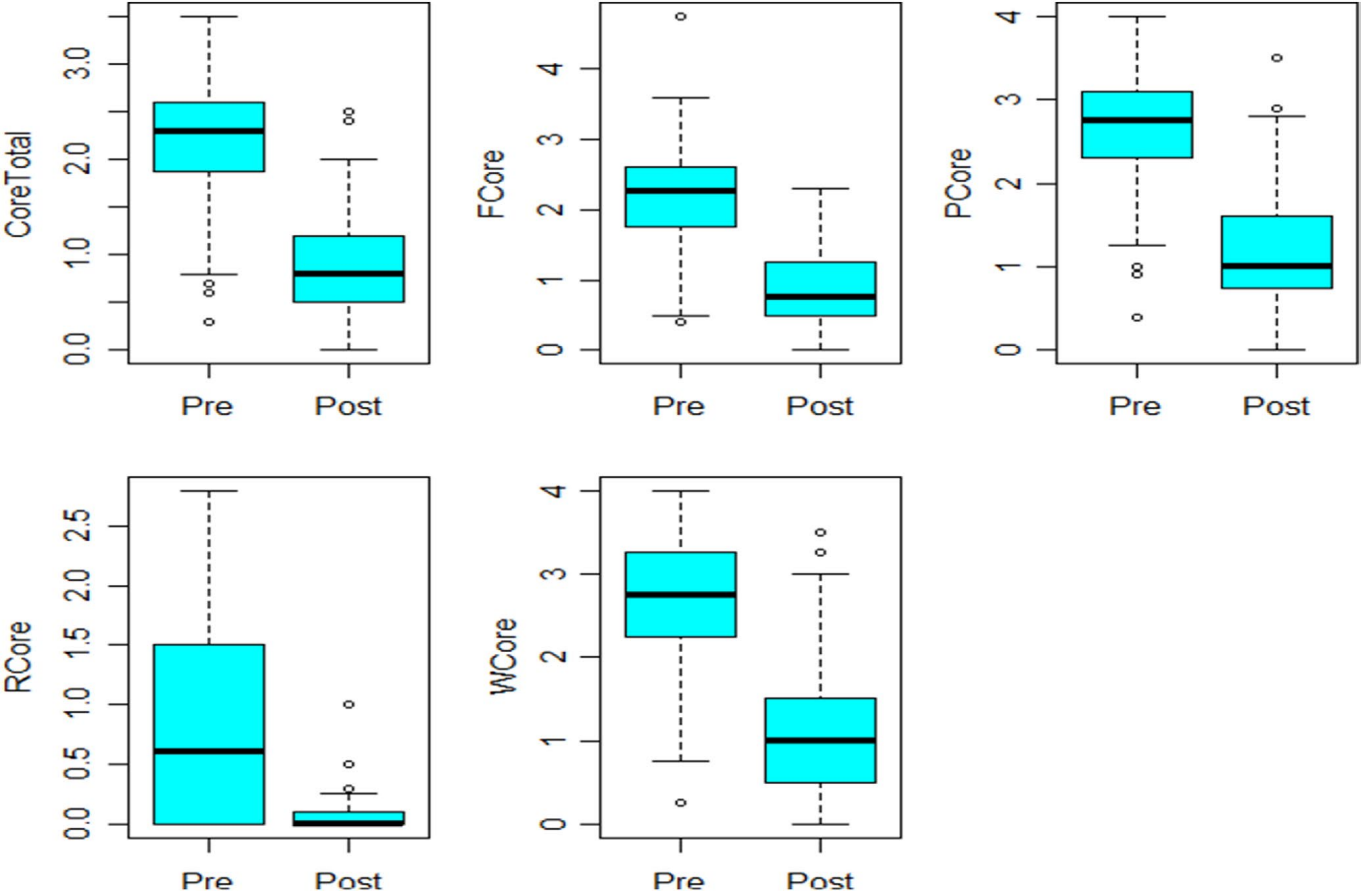
Box plots for the primary care data.

The primary dataset contained 100 observations and 11 variables. This dataset refers to primary care participants who accessed psychosocial assessment and intervention directly from GP‐led services without attending local CMHT services. For each of the pre variables, three observations were missing, and for each post‐variable, 42 observations were missing. Figure 1 presents the box plots of the primary data for pre‐ and post‐measurements for all the five variables. For all the variables, there were decreases between pre and post‐measurements. Also, from the figure, several mild outliers can be noticed for all the variables, which were retained for analyses.

The paired t‐test was conducted for the variables CORE total, F CORE, P CORE and W CORE, and all variables demonstrated statistical significance at the 5% level, as indicated by the *p*‐values in Table 3. Specifically, for the variable Core total, there was a significant mean decrease of 1.16 between pre‐intervention and post‐intervention measurements (*p* < 0.001), indicating a reduction in distress. As the variable R core did not meet the normality assumptions, the Wilcoxon test was used to compare the distribution of pre‐ and post‐intervention measurements (Rosner, Glynn, and Lee 2006). The results from the Wilcoxon test also showed a significant difference in the distribution of pre‐ and post‐measurements for the variable R core (*p* < 0.001). These findings suggest that the intervention led to significant improvements in the measurements of Core total, F core, P core and W core. The small *p*‐values further support the significance of these improvements.

**TABLE 3:** Descriptive statistics for the primary care.

	Pre, mean (SD) *n = 97*	Post, mean (SD) *n = 58*	Mean difference [95% CI]	Test statistic	*p* value
Core total	2.14 (0.63)	0.89 (0.53)	1.16 [1.01, 1.31]	*t*_57_ = 15.4	< 0.001
*F* core	2.138 (0.77)	0.88 (0.56)	1.18 [1.00, 1.38]	*t*_57_ = 12.2	< 0.001
*P* core	2.63 (0.71)	1.18 (0.71)	1.39 [1.19, 1.58]	*t*_57_ = 14.3	< 0.001
*W* core	2.73 (0.82)	1.21 (0.82)	1.51 [1.29, 1.74]	*t*_57_ = 13.6	< 0.001
*R* core	0.60 (1.50)	0.00 (0.10)			< 0.001

*Note:* Mean (SD) reported for normally distributed variables and Median (IQR) for non‐normal variables (*R* core). This table presents the summary statistics of pre and post. The paired t‐test was done if the normality assumption was met, and the Wilcoxon rank sum test was done otherwise (for the variable R core).

### 5.2 Secondary Care Data Set

**FIGURE 2:**
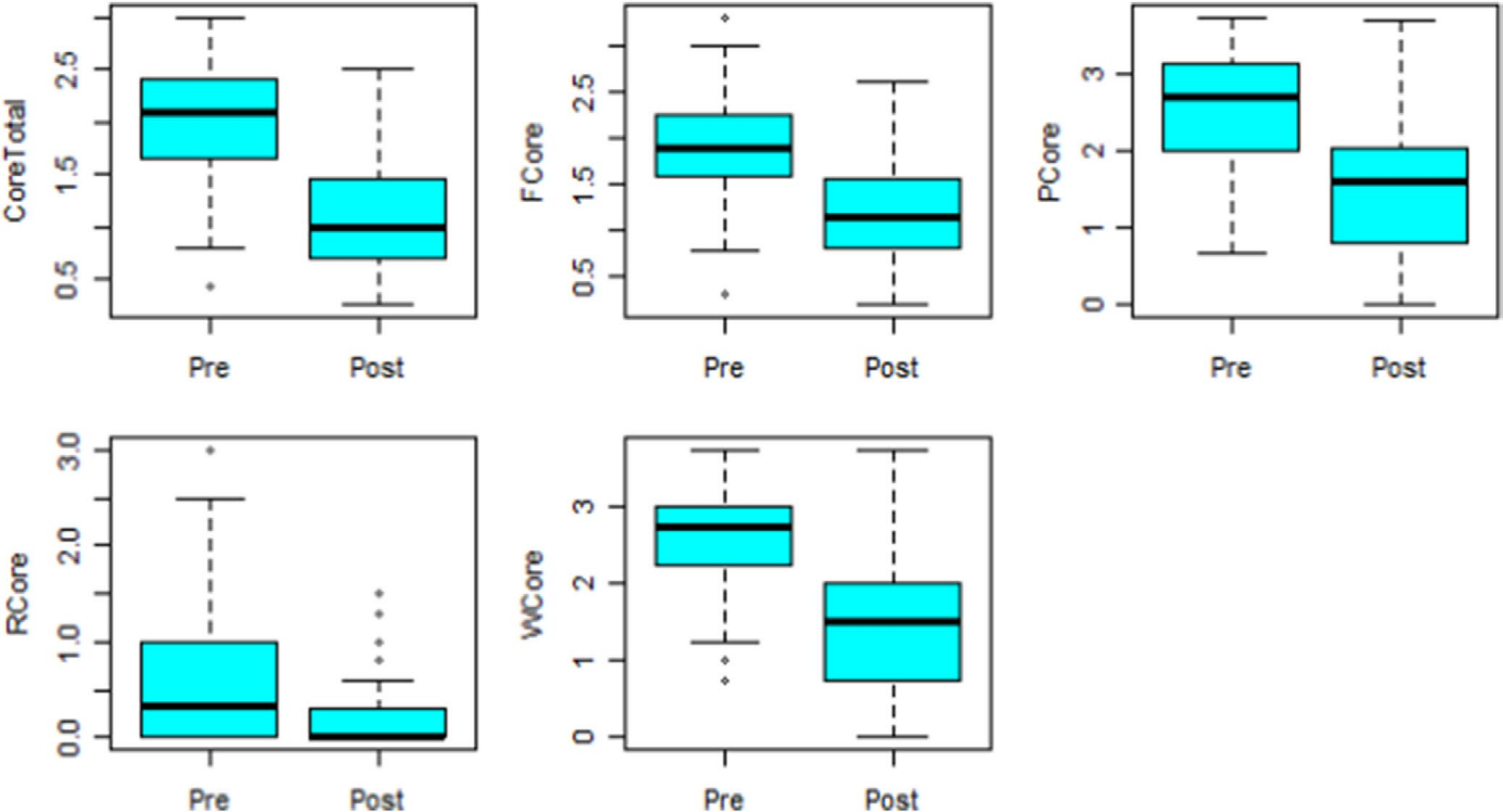
Box plots for the secondary care data.

This dataset refers to data obtained in the secondary care setting. That is, participants who accessed psychiatric assessment from secondary care consultant psychiatry. The secondary care dataset contained 38 observations and 11 variables, similar to the primary dataset, there were also missing data points in the secondary dataset. Three observations were missing for all the pre‐variables, 10 observations were missing for Post F Core and 11 observations were missing for all the other post‐variables. Figure 2 presents the box plots of the data for pre‐ and post‐measurements for the five variables. For all the variables, the difference between pre‐ and post‐measurements also decreased. Further, from the figures, several mild outliers can be noticed in the variables core total, F core and R core.

Table 4 presents descriptive statistics for the secondary care data and for all the variables except R core. The paired t‐test was used to determine if there are significant mean differences in pre‐ and post‐intervention measurements. From the results, there is a significant mean difference in the pre‐ and post‐measurements with *p* < 0.001 for all the four variables. The variable R core was not normally distributed and thus the Wilcoxon test was used instead. The Wilcoxon test is advantageous as it is non‐parametric and does not require any distributional assumptions. For the R core variable, the Wilcoxon test revealed that there was a significant difference in the distribution of the pre‐ and post‐measurements with *p* = 0.003.

**TABLE 4:** Descriptive statistics for the secondary care.

	Pre, mean (SD) *n = 38*	Post, mean (SD) *n = 28*	Mean difference [95% CI]	Test statistic	*p* value
Core total	1.97 (0.63)	1.16 (0.65)	0.76 [060, 0.91]	*t*_26_ = 9.9	< 0.001
*F* core	1.89 (0.59)	1.22 (0.63)	0.66 [0.48, 0.84]	*t*_27_ = 7.4	< 0.001
*P* core	2.52 (0.84)	1.54 (0.98)	0.95 [0.75, 1.15]	*t*_26_ = 9.7	< 0.001
*W* core	2.62 (0.77)	1.57 (0.96)	1.07 [0.81, 1.34]	*t*_26_ = 8.4	< 0.001
*R* core	0.33 (1.00)	0.00 (0.30)			0.003

### 5.3 Decider Skills Group Data

**FIGURE 3:**
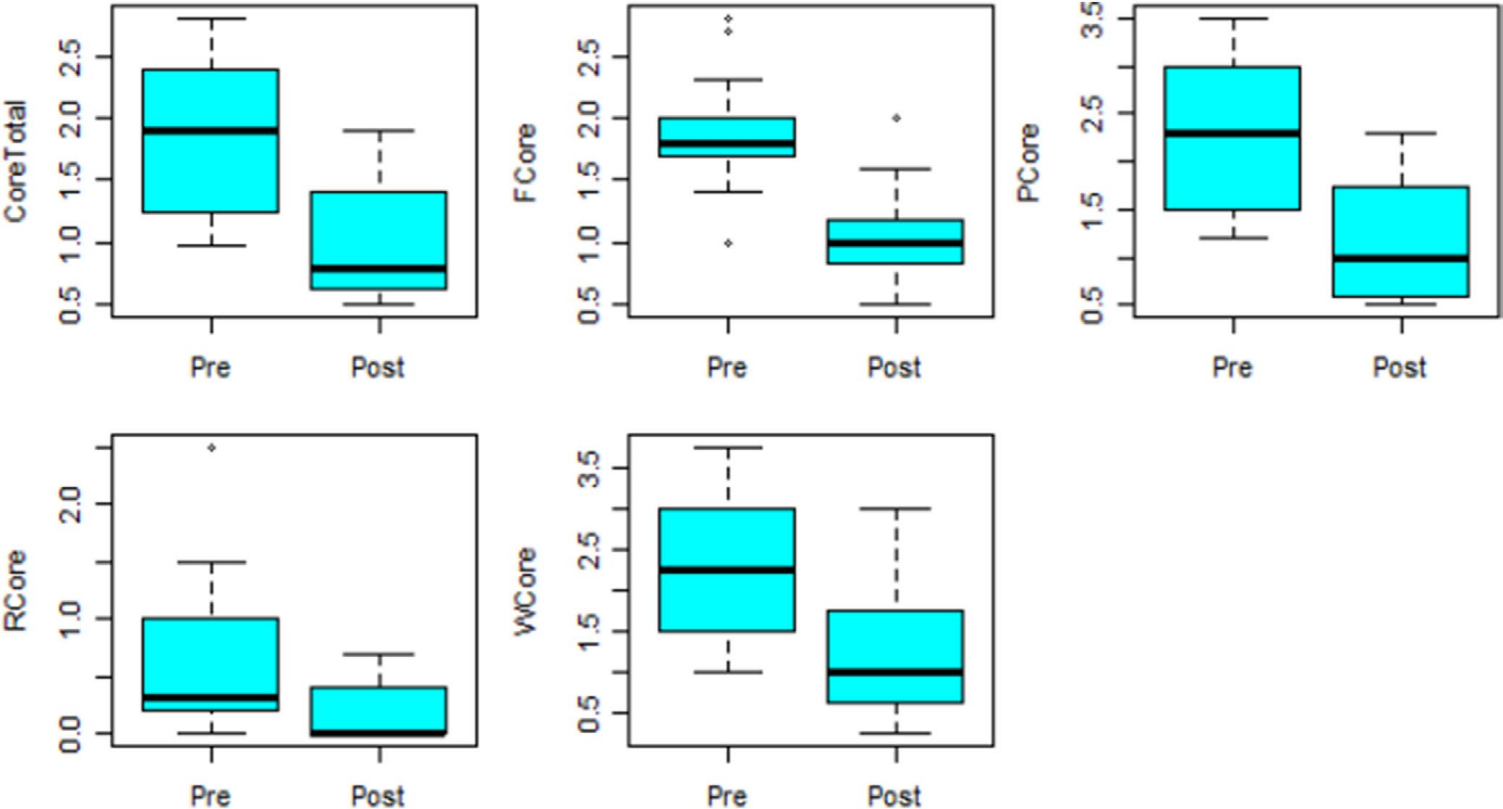
Box plots for the groups data.

The group data refers to group Decider Skills (participants who accessed group Decider Skills from both Primary and Secondary Care). There were three groups provided in line with COVID‐19 restrictions which were in place at the time. The group dataset had 19 observations and 11 variables. For each of the pre‐variables, two observations were missing, and for each of the post‐variables, four observations were missing. The Figure 3 presents box plots for the group dataset. From the visualisation, there seems to be outliers in the variables F core and R core. The differences across pre‐ and post‐measurements seem to clearly be visible.

Table 5 presents the descriptives for the groups data. The paired t‐test was undertaken for all the variables except R core because it was not normally distributed. For all the variables, there is a significant mean difference in the pre‐ and post‐measurements as with *p* < 0.001 for all the variables, it is indicated that the intervention was effective. For the variable R core, the Wilcoxon test was used, and the analysis revealed a significant difference in the distribution of the pre‐ and post‐measurements.

**TABLE 5:** Descriptives of the group data.

	Pre mean (SD) *n = 17*	Post mean (SD) *n = 15*	Mean difference [95% CI]	Test statistic	*p* value
Core total	1.88 (0.58)	0.99 (0.46)	0.88 [0.57, 1.18]	*t*_14_ = 6.1	< 0.001
*F* core	1.89 (0.44)	1.08 (0.45)	0.79 [0.47, 1.11]	*t*_14_ = 5.3	< 0.001
*P* core	2.303 (0.77)	1.19 (0.63)	1.12 [0.76, 1.49]	*t*_14_ = 6.6	< 0.001
*W* core	2.279 (0.92)	1.28 (0.81)	1.03 [0.65, 1.41]	*t*_*1*4_ = 5.8	< 0.001
*R* core	0.33 (0.80)	0.00 (0.40)			0.003

### 5.4 Additional Performance Indicators

The average waiting time for psychosocial assessment, CBT or Decider Skills through PARC was 3.6 working days. 97% (*n* = 208) of participants were offered an initial appointment within 1 week or less from the time of self‐referral. 2% (*n* = 4) were seen within 2 weeks and the remaining 1% (*n* = 3) were seen within 3 weeks. Service users presenting with mild to moderate mental health diagnoses, and attending the secondary care service, have reduced in number from 50% to 28% of the overall service caseload since the commencement of the PARC referral pathway. This potentially increases access to secondary care psychiatric assessment and interventions for service users experiencing severe and enduring mental health illness.

## 6 Discussion

In implementing PARC, our aims were to improve service user outcomes, bolster access to mental health creating integrated care and reduce mild to moderate presentations within the secondary care service. This study illustrates that service users benefitted from PARC and demonstrated improvements across all CORE‐OM items including well‐being, problems and symptoms, functioning and risk. Access to care revealed an average waiting time for assessment and interventions being 3.6 working days. Regrettably, due to waiting list data being aggregated across community mental health teams, we were unable to compare access to care pre and post the implementation of PARC. Favourable waiting times compared to those described by Mental Health Reform (2022) were evident. A majority of service users referred to PARC were seen within 1 week or less. This is positive in the context of Irish national policy, Sláintecare, which seeks a waiting time of no more than 12 weeks (Government of Ireland 2017). In contrast, Punton, Dodd, and McNeill (2022) report waiting lists of 18 weeks or longer to access mental health services in the United Kingdom being commonplace, with wait times of up to 10 weeks reported in the United States (Sun et al. 2023). Our study implies the potential for mental health RANP‐led services to impact positively on access to care, a benefit also reported by Htay and Whitehead (2021) and Moxham, McMahon‐Parkes (2020).

The motivation for developing PARC stems from a view that the needs of service users experiencing mild to moderate mental health illness in primary care could be augmented. This is because such service users may not meet criteria for psychiatry referral, however, primary care interventions may not be available, may have lengthy waiting times or may be experienced as insufficient, as discussed by Moise, Navin, and Wainberg (2021). Additionally, primary care service providers may perceive that the service user's level of need exceeds their capacity, particularly noted by Wlodarczyk et al. (2018), in the case of borderline personality disorder. The results of our study support a view that PARC is associated with improvements in mental state for service users, as demonstrated by changes in the CORE‐OM scores. Improvements were seen across all variables—well‐being; problems and symptoms; and functioning and risk.

The lack of clarity concerning the development and implementation of RANP roles across Europe is concerning (De Raeve et al. 2024). Our study adds valuable details regarding the implementation of a RANP role in the Republic of Ireland. Challenges in the implementation and evaluation of RANP practice have been apparent for over 20 years (Bryant‐Lukosius et al. 2004). Only recently have frameworks for the evaluation of advanced practice roles been developed in other European countries (Unsworth et al. 2022). It is beyond time that RANPs articulate their role, their collaboration with other healthcare professionals and the value added within the health service (Thompson and McNamara 2022).

From an implementation perspective, there are several factors to consider. Firstly, as Pawson (2006) points out, local idiosyncrasies must be taken into account, so that PARC (or any RANP‐led intervention) can be replicated elsewhere. The community mental health service in this study had an established tradition of investing in psychological and social therapies. These include making available opportunities for postgraduate CBT study and Decider Skills. The importance of such approaches is well evidenced in mental health contexts (Lamboy et al. 2022; Hurley et al. 2022). Furthermore, in their seminal work, Mazmanian and Sabatier (1989, 279) refer to ‘veto points’. These occur when the implementing agency is given extensive powers but must work through other government departments and agencies, resulting in significant barriers potentially being created. However, in the Irish context, national policy is heavily weighted in favour of RANP expansion (Department of Health 2019). Nonetheless, role acceptance and visibility are important if implementation is to succeed (Evans et al. 2020) with both factors apparent in the case of PARC.

In exploring implementation theory and nursing, Bergen and While (2005) suggest that clarity, concordance with espoused nursing values, local practice and the personal proclivities of the individual nurse impact significantly on how policy is implemented. The PARC RANP‐lead and now additional candidate advanced nurse practitioner expressed a deeply held belief in the importance of stakeholder communication, and the ability of psychosocial assessment and brief psychosocial interventions to improve service user outcomes. PARC was characterised by positive interactions within the complex system that is a mental health service. Senior and local nursing leadership support was apparent, the importance of which is emphasised by Salmela, Koskinen, and Eriksson (2016) and Duignan, Drennan, and McCarthy (2024), while interdisciplinary team members, in particular, consultant psychiatrists invested in the approach. A shared belief in the importance of psychosocial interventions in improving service user outcomes, and access, motivated a team of healthcare professionals to fully engage in this RANP‐led initiative. Evidence suggests that RANPs exert clinical autonomy and are prepared to step up and establish new services, in partnership with other professions (Levy‐Malmberg et al. 2024).

The impact of PARC in providing full episodes of care is important especially in light of the limited evidence for advanced nurse practitioner delivered interventions for mental health illness in primary care (Halcomb et al. 2019). Furthermore, PARC has the potential to be cost effective and provide alternatives to more intrusive forms of care, such as hospitalisation, which are characteristics of RANP roles reported upon by Liu et al. (2020). As noted, access to care was viewed as efficient. Seventy‐three per cent or 157 of those service users referred to PARC attended and completed interventions. The rate of non‐completion is similar to other studies exploring mental health nursing interventions at the interface of primary and secondary care. For instance, Kenwright et al. (2024) in a UK study, report that while 83% of first treatment appointments were attended, 25% of service users discontinued after a first treatment appointment. Based on our results, it is proposed that PARC has characteristics in keeping with the key objectives of the CPG *Common Mental Health Problems: Identification and Pathways to Care*, developed by NICE (2011), which was further reviewed in 2018. PARC provides locally based effective assessment with interventions. There is a direct pathway for the RANP to refer to psychiatry where required, with a clearly delineated clinical supervision process.

### 6.1 Strength and Limitations of the Work

While a validated measurement tool is utilised, this is an uncontrolled study that observes differences in clinical presentation between time points, which impacts what conclusions may be drawn. Furthermore, there are limitations related to generalisability of results pertaining to a single study, in a single location, as particular personnel and organisational factors may not be replicated elsewhere. At present, there is no longitudinal follow up post‐intervention. While the CORE‐OM was completed confidentially by the service user alone, data may be affected by acquiescence bias. Data were collected during the COVID‐19 pandemic, and this affected participation rates and completed post‐intervention measures. While steps have been taken to mitigate this, it is a limitation. As discussed previously, it was not possible to compare pre‐ and post‐waiting times due to the aggregation of community mental health teams waiting list data. Finally, while the interventions utilised in PARC have a track record within mental health service provision, there is variability in how nursing interventions in primary care are understood and implemented across settings.

The strengths of this study include that it reports real‐world initiatives conceptualised, led and delivered by a nursing team inclusive of clinicians and managers at various grades of seniority, in collaboration with psychiatry. It addresses the interface between secondary and primary care, which is an area targeted for improvements in access and intervention in national and international policies. The study uses a validated scale (Barkham et al. 2006; Evans et al. 2002) which demonstrates abatement of symptoms of mental health illness, with evidence of improvements in all CORE‐OM items. Appropriate statistical tests have been used to address missing data. Certain opportunities and challenges in implementation are alluded to, which may inform other mental health services in planning similar programmes of care. In particular, this may add to the understanding of the potential for advanced nursing practice.

### 6.2 Recommendations for Further Research

Further research into the precise factors which enhance implementation of RANP initiatives at the mental health primary and secondary care interface is required. This may allow for a better understanding of how strategy and integrated healthcare may flourish (Carney 2024). In relation to PARC itself, this study suggests that service user clinical outcomes were improved by its introduction. Additional controls, and longitudinal follow‐up is merited to further understand the impact of this intervention. Specifically, studies exploring measures before and after implementation of such RANP services across different settings, and possibly including control/treatment as usual comparators would be useful in order to demonstrate the effect of services similar to PARC being implemented.

### 6.3 Implications for Policy and Practice

Advanced practice nurses, an example being PARC, provide a cost‐effective solution to improving clinical outcomes for overburdened primary and secondary healthcare services (Mackavey et al. 2024). From an implementation perspective, it is critical to establish collaborative relationships with other healthcare professionals, ensuring team consensus while fostering integration between primary and secondary care providers, as recommended by Torrens et al. (2020). Consequently, it is important that RANPs receive tuition in implementation science as part of their initial formation. Knowledge of a scientific implementation framework supported PARC. Initiatives such as PARC may also create additional capacity within secondary care mental health services for those living with severe and enduring mental health illnesses. As such, development of a model of care for mental health advanced nurse practitioners at the interface of primary and secondary care settings may be merited.

### 6.4 Conclusion

The intervention was associated with improvements in mean differences between pre‐ and post‐intervention measurements of the CORE—OM variables and total. The paired t‐tests for these variables resulted in *p* < 0.001, implying statistically significant decreases. The variable R Core, was therefore analysed using the Wilcoxon test in all data sets—again showing a significant reduction in post‐measurements, further supporting the effectiveness of the intervention. These findings are consistent with results of systematic reviews of RANP led initiatives (Htay and Whitehead 2021; Swan et al. 2015) describing these as cost‐effective, improving service user outcomes and enhancing service related outcomes. PARC has resulted in more accessible care for people with mild to moderate mental health illnesses from primary care, and those with severe and enduring mental health illness in secondary care. From an implementation perspective, proactive engagement with stakeholders, clear communication and identifying barriers promptly were key. Therefore, it is proposed that services based on similar models to PARC may be successfully implemented elsewhere with benefits to service users. Moreover, it is proposed that RANP roles can exert a positive impact at the interface between primary and secondary care. The potential for further integration is apparent, as suggested by Damien et al. (2024), with the potential for improved resource utilisation and service user outcomes.

## Conflicts of Interest

The authors declare no conflicts of interest.

## Peer Review

The peer review history for this article is available at https://www.webofscience.com/api/gateway/wos/peer‐review/10.1111/jan.16498.

## Supporting information


Appendix S1.

## Data Availability

Data are held by the corresponding author and may be reviewed on request. All data associated with this manuscript has been lawfully acquired.
